# Health risks assessment of polycyclic aromatic hydrocarbons (PAHs) in meat kebabs through meta-analysis and Monte Carlo simulation

**DOI:** 10.1016/j.mex.2025.103502

**Published:** 2025-07-12

**Authors:** Fathollah Gholami-Borujeni, Fatemeh Mortezazadeh, Somayeh Hoseinvandtabar, Anoushiravan Mohseni-Bandpei, Hadi Niknejad

**Affiliations:** a Department of Environmental Health Engineering, Health Sciences Research Center, Mazandaran University of Medical Sciences, Sari, Iran; b Department of Environmental Health Engineering, School of Public Health, Tehran University of Medical Sciences, Tehran, Iran; c Student Research Committee, Department of Environmental Engineering, School of Public Health and Safety, Shahid Beheshti University of Medical Sciences, Tehran, Iran; d Environmental and Occupational Hazards Control Research Center, Shahid Beheshti University of Medical Sciences, Tehran, Iran

**Keywords:** Global analysis, Health risk assessment, PAHs, Monte Carlo simulation (MCS), Meat kebabs

## Abstract

Polycyclic aromatic hydrocarbons (PAHs) are included in various everyday food products, prompting worries over their possible detrimental effects on human health. This study aimed to estimate the health risks associated with PAH contamination in meat kebabs using a comprehensive meta-analysis and probabilistic risk assessment framework. The researchers collected information on PAH levels in meat kebabs (cooked by barbecuing, grilling, broiling, or smoking) from 42 studies found in well-known databases like Scopus, PubMed, Science Direct, Google Scholar, and Web of Science, covering the years from 2011 to October 2023. Furthermore, health risks were assessed through chronic daily intake (CDI), hazard quotient (HQ), and incremental lifetime cancer risk (LTCR), with uncertainty analyzed utilizing Monte Carlo simulation (MCS, 10,000 iterations). The investigation revealed that meat kebabs were made using barbecuing (7.4 %), broiling (11.2 %), smoking (25.7 %), and grilling (55.7 %) techniques. The highest average concentrations were observed for pyrene (Pyr: 359.32 µg/kg) and naphthalene (Nap: 263.20 µg/kg), while benzo[a]pyrene exhibited a lower mean concentration (BaP: 1.79 µg/kg). Risk assessment indicated that HQ values for some PAHs, particularly BaP (1.× 10²) and Nap (3.93), exceeded the threshold of 1, indicating possible non-carcinogenic effects at high intake levels. However, LTCR values for all PAHs remained below 1 × 10⁻⁶, indicating negligible lifetime cancer risk according to USEPA guidelines. According to the results, although meat kebabs may pose a non-carcinogenic concern at elevated intake levels, the lifetime cancer risk appears within acceptable limits. These findings highlight the importance of promoting safer cooking techniques and public awareness to mitigate exposure to PAHs through dietary sources.

## Highlights

• We evaluate the presence and levels of 16 priority PAHs in meat kebabs from specific original articles from 2011–2023.
• The meta-analysis method was used to examine the levels of PAHs.
• We evaluate risk assessment of both carcinogenic and non-carcinogenic effects posed by PAHs using MCS method.

## Graphical abstract

**Figure:**
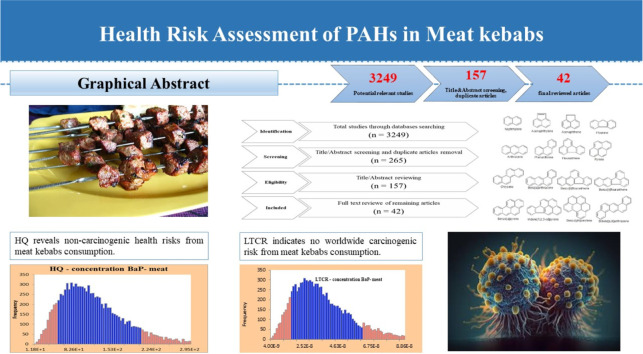

**Specifications table**

**Table:** 

**Subject area**	
**More specific subject area**	PAHs in Meat kebabs
**Name of the reviewed methodology**	Meta-Analysis and Monte Carlo Simulation
**Keywords**	Global Analysis, Health Risk Assessment, PAHs, Monte Carlo simulation (MCS), Meat kebabs
**Resource availability**	like Scopus, PubMed, Science Direct, Google Scholar, and Web of Science
**Review question**	Investigating the presence and levels of 16 priority PAHs in barbecued meat from specific original articles from 2011–2023 • Investigating PAH levels using a meta-analysis approach • Assessing the risk of carcinogenic and non-carcinogenic effects of PAHs using the MCS approach

## Background

PAHs are organic chemicals consisting of two or more fused aromatic rings. They exist in different isomeric configurations and as numerous environmental compounds [1,2]. PAHs represent a considerable risk to human health, as numerous compounds exhibit carcinogenic, mutagenic, and genotoxic properties [3]. These harmful chemicals are fat-soluble and not easily biodegradable and hence tend to accumulate in living organisms by bioconcentration and biomagnification processes. Therefore, there is a potential health risk to human beings through dietary intake [4,5]. The SCF (Scientific Committee for Food), JECFA (Committee for Food Additives), and EFSA (European Food Safety Authority) have evaluated PAH compounds in food, identifying 16 specific PAHs, including Dibenzo[a,h]anthracene (DahA), Phenanthrene (Phe), Benzo[b]fluoranthene (BbF), Benzo[a]pyrene (BaP), Indeno[1,2,3-cd]pyrene (IcdP), Chrysene (Chr), Acenaphthene (Ace), Benzo[ghi]perylene (BghiP), Naphthalene (Nap), Fluoranthene (Flu), Acenaphthylene (Acy), Anthracene (Ant), Fluorene (Fle), Benzo[a]anthracene (BaA), Benzo[k]fluoranthene (BkF), and Pyrene (Pyr) [6]. However, for the purposes of this study, only eight of these compounds (BaP, Flu, Anth, Ace, Acy, Pyr, Flrt, and Nap) were selected for quantitative risk assessment. This decision was based on [1] the consistent availability of concentration data for these eight PAHs across the 42 studies included in our meta-analysis, and [2] the availability of toxicological benchmarks such as cancer slope factors (CSFs) and reference doses (RfDs) provided by the U.S. Environmental Protection Agency (USEPA) and EFSA, which are essential for conducting probabilistic risk assessments.

The contamination of the cooked food may either come from the raw food or from the cooking process. The formation of PAHs during meat processing is influenced by several variables, including cooking temperature, duration, fuel type, meat composition (particularly fat content), and the distance between the heat source and the food [2]. High-temperature methods such as direct charcoal grilling are particularly conducive to PAH generation due to fat combustion and smoke deposition. These factors must be considered when evaluating health risks from grilled meat products [[6], [7], [8]]. PAHs are frequently detected in meat, oils, and fats, as well as in smoked and non-smoked meat products (including meat, fish, and oysters). Studies conducted on meat have identified >30 different types of PAHs in cooked meat, with 15 of them being linked to the development of cancer. The acceptable thresholds for BaP and ∑PAHs (including BaA, BaP, Chr, and BbF) in meat and meat products are set at 5 and 30 μg/kg, respectively [9]. According to the Joint FAO/WHO (food and agriculture organization/world health organization) Expert Committee on Food Additives (JECFA), BaP can serve as a marker for genotoxic and carcinogenic PAHs found in food [10]. The aim of this study was to investigate PAHs in meats using systematic review and meta-analysis. Additionally, the MCS was applied to estimate potential carcinogenic and non-carcinogenic health risks associated with dietary exposure to PAHs in meat kebabs [11].

## Method details

### Materials and methods

#### Search method

This study carried out an extensive review of articles between the years 2012 and October 2023, examining the correlation between PAHs and different cooking techniques, namely grilling, broiling, smoking, and barbecuing. A comprehensive review of the literature was done on different databases, namely Google Scholar, Science Direct, PubMed, Scopus, and Web of Science. Different keywords were utilized to have an extensive coverage of the subject. The search terms broiled meat, grilled meat, barbecued meat, and smoked meat were used in combination with the term PAHs. In order to have a systematic and transparent search process, the researchers meticulously followed the PRISMA guidelines [12]. The study delineated precise inclusion criteria for qualifying papers, comprising the following aspects: Firstly, the study's inclusion requirements required the article to be published between 2011 and October 2023. Secondly, all investigations assessed the presence of at least one polycyclic aromatic hydrocarbon in meat. Thirdly, the article provides an explanation of how to detect PAH concentrations in various types of meat, including barbecued, grilled, broiled, and smoked meat. Fourthly, the article's full text is readily available. To ensure the fulfillment of the inclusion criteria, two authors independently evaluated the papers. In the event of any discrepancies, a third author was consulted to reach a resolution. The collected data from the final articles encompassed several aspects, such as the type of meat, concentration (range and mean ± SD), heat source (direct or indirect heat), number of samples, types of PAHs, and year of publication.

#### Data acquisition and meta-analysis

**Fig. 1:**
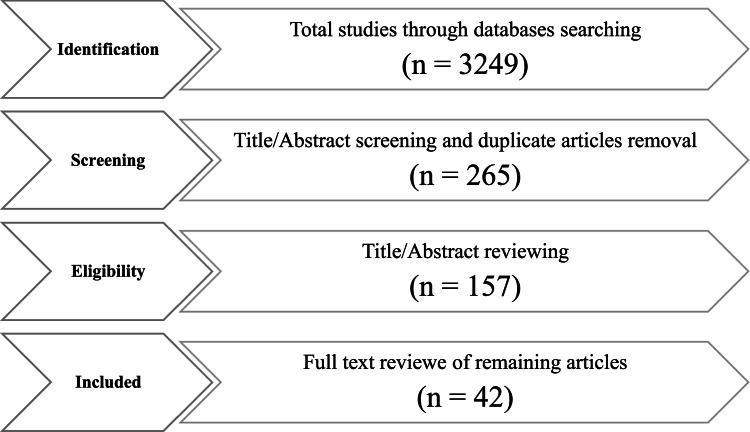
comprehensive flow diagram of the literature review.

The systematic review methods were utilized by the meta-analysis to estimate meat product PAH levels. The analysis comprised 42 original studies with an aggregate sample size of 12,307 (Fig 1). According to the matrix, the various samples were selected as two separate groups: 1) Carcinogenic group and 2) non-carcinogenic group. The PAH content in various kinds of meat was assessed using Eq. (1).

(1) SE = SD/N^0.5^

This equation takes into account variables such as the sample size (N), standard deviation (SD), and standard error (SE) of the PAH concentration in meat [13]. To assess the heterogeneity among studies and their outcomes, the I^2^ and Q tests were employed, which provide insights into the variation present in the data.

#### Health risk assessment

In this study, we made an extensive assessment of the possible human health risks due to the ingestion of PAH-containing meat. To predict both the non-carcinogenic and carcinogenic risks, we used Eqs. (2)-(4) to evaluate the daily intake and the corresponding risks [14].

(2) $$\text{CDI}=\frac{C\;\times \;\text{CF}\;\times \;\text{EF}\;\times \;\text{ED}\;\times \;\text{IR}\;\times \;\text{RPF}\;}{BW\;\times \;\text{AT}}$$

where CDI represents the Chronic daily intake of PAHs (mg/kg.day), C denotes the concentration of PAHs in meat (μg/kg), CF is the conversion factor (10^−6^ kg/mg), EF is the exposure frequency (d/y), ED represents the duration of exposure in years, IR is the daily intake or ingestion rate of meat (0.116 g/n-day), RPF denotes the relative potency factors, BW represents the average body weight (77.45 kg), and AT denotes the average exposure time (days) which is 365 *×* 54 for non-carcinogenic risk and 365 *×* 70 for carcinogenic risk.

(3) $$\text{HQ}=\frac{\text{CDI}}{\text{RfD}}$$

Where CDI represents the daily chronic consumption, RfD means a reference dose (mg/kg/day) and HQ means hazard quotient. The HQ value <1 (HQ < 1) indicates no adverse effects, while the HQ value greater than 1(HQ > 1) indicates non-carcinogenic adverse health effects.yuy

(4) LTCR = CDI *×* CSF

where LTCR is the excess lifetime cancer risk and CSF is the cancer slope factor (mg/kg/day)^−1^ [[14], [15], [16]]. Table 1 displays the RfD values and other pertinent parameters associated with the exposure to PAHs through meat kebab consumption. Numerous PAHs have been classified by IARC as group 1 carcinogens, signifying their established capacity to induce cancer in humans. The USEPA (United States Environmental Protection Agency) has estimated the CSF value for BaP, which is one of the most extensively researched PAHs, to be approximately 7.3 (mg/kg/day)^−1^ [[17], [18], [19]]. Studies are ongoing to ascertain the CSFs of different PAHs found in meat and other food items, such as chicken kebabs. It's important to note that the CSFs could be different for different PAHs. To come up with effective ways to lower PAH contamination and encourage healthier eating, we would need a full picture of the levels and likely health risks of these compounds. Thus, researchers are actively working to determine CSFs for other PAHs present in meat and other food products [20].

**Table 1:** parameters for estimating carcinogenic risk and non-carcinogenic risk.

Exposure factors	Unit	Values
IR	g/n-day	0.116
EF	days/year	365
ED	years	54
AT for carcinogens	days	25,550
AT for non-carcinogens	days	19,710
BW	kg	77.45
RPF	No Unit	BaP=1, Anth = 0.01, Ace =Acy = Pyr = Flrt = flu = Nap = 0.001
RfD	mg/kg/day	BaP=0.003, Anth = 0.3, Ace = Acy = 0.06 Pyr = 0.03, Flrt = flu = Nap = 0.04

#### Statistical analysis

This study utilised the MCS technique, augmented with the Oracle Crystal Ball® ribbon (version 11.1.3, Oracle, Inc., USA), to evaluate the possible health risks to humans associated with PAHs in meat kebab consumption. The MCS method is a strong statistical approach used to figure out the range and likelihood of different outcomes by running a lot of simulations. In the current research, the MCS technique was used to carry out a comprehensive analysis with the purpose of estimating the possible range of both non-carcinogenic and carcinogenic risks due to PAH exposure in meat. The study utilised the MCS method by conducting 10,000 trials to precisely assess the spectrum of potential risks linked to PAH exposure. Scientists could figure out how likely different outcomes were to happen using this computational technique, and they were able to accurately predict the range of risk calculations, even though the data was not completely clear. The findings derived from the MCS were pivotal in designing efficient risk management measures to reduce the potential health risks linked to PAH exposure in meat [11,21].

## Results

### Features of research and literature search

The identification of the relevant research articles in this study was accomplished by utilizing a targeted database consisting of 3249 original articles. After carefully reviewing the identified research studies, any articles that were deemed irrelevant or redundant were meticulously excluded from the analysis. After conducting a thorough process of eliminating duplicate articles and carefully screening the title/abstract, a total of 2984 irrelevant articles were filtered out, leaving only 265 articles. After the process of eliminating duplicate articles and screening the titles and abstracts, any articles lacking the desired information were subsequently removed, resulting in a total of 42 articles that remained for the meta-analysis (Fig 1). The results of this study indicate that the most widespread kinds of meat products are grilled meat (55.7 %), smoked meat (25.7 %), broiled meat (11.2 %), and barbecued meat (7.4 %). The examination of the sources of heat used to cook indicates that a vast majority of meats, representing 63.2 %, were cooked by charcoal heat, whereas 36.8 % were cooked by gas heat. Furthermore, it was observed that 40.8 % of the meats were heated indirectly, while the remaining 59.2 % were heated directly.

The results of this study show that, after analyzing the methods used to quantify the levels of PAHs in meat, 33.4 % of the studies used HPLC, 59.2 % used gas chromatography (GC), and 7.4 % used other methods. It is worth noting that these methods are widely adopted for assessing PAH levels in food samples and are known to yield precise and dependable results.

### Meta-analysis

The results of the study are clearly shown in Table 2, which gives a thorough analysis of the concentration of 16 PAHs in meat samples. These samples were collected by using various detection methods, subjected to various heat sources, and with different sample sizes to give a thorough assessment. The table presents essential information, including the mean, standard deviation, and the minimum and maximum concentration levels identified in extensive global research studies. The concentrations of these PAHs were carefully analysed using a variety of analytical techniques, including HPLC and GC. These analyses were conducted on meat samples that were prepared carefully under a variety of heat sources, including gas and charcoal, and a variety of heating processes, including direct and indirect heating processes.

**Table 2:** Meta-analysis Concentration of 16 PAHs in meat kebabs.

**No**	**PAHs type**	**Detection Methods**	**Type of Hate**	**Number of samples**	**Mean (µg/kg)**	**SD**	**Lower concentration (µg/kg)**	**Upper concentration (µg/kg)**
1	Acenaphthylene (Acy)	HPLC-FLD^1^/ GC–MS^2^	Charcoal/gas	488	23.470	0.1773	0.014	180.77
2	Benzo[a]Pyrene (BaP)	HPLC-FLD/ GC–MS/ HPLC-DAD^3^	Charcoal/gas	1440	1.78690	0.3921	ND^4^	54.58
3	Bbenzo[b]Fluoranthene (BbF)	HPLC-FLD/ GC–MS/ HPLC-DAD	Charcoal/Gas	72	4.7954	0.3723	ND	34.45
4	Benzo[k]Fluoranthene (BkF)	HPLC-FLD/ GC–MS	Charcoal/grill	948	2.4308	0.1259	ND	42.83
5	Benzo[ghi]Perylene (BghiP)	HPLC-FLD/ GC–MS/ HPLC-DAD	Charcoal/grill	951	3.4008	0.1050	ND	124.79
6	Fluorene (FLU)	HPLC-FLD/ GC–MS/ HPLC-DAD	Charcoal/Gas	597	93.5384	4.38535	0.0030	500.02
7	Chrysene (Chr)	HPLC-FLD/ GC–MS/ HPLC-DAD	Charcoal/Gas	1316	53.2644	33.1462	0.008	1309
8	Benz[a]anthracene (BaA)	HPLC-FLD/ GC–MS/ HPLC-DAD	Charcoal/grill	1326	32.5631	20.3880	0.001	99
9	Dibenzo[a,h]Anthracene (DBahA)	HPLC-FLD/ GC–MS/ HPLC-DAD	Charcoal/grill	966	3.9630	0.1124	ND	163.31
10	Indeno[1,2,3- cd]pyrene (Ind)	HPLC-FLD/ GC–MS/ HPLC-DAD	Charcoal/grill	957	4.1606	0.1755	ND	106.94
11	Naphthalene (Npb)	HPLC-FLD/ GC–MS/ HPLC-DAD	Charcoal/grill	247	263.2024	8.0711	0.004	1940
12	Phenanthrene (Phen)	HPLC-FLD/ GC–MS/ HPLC-DAD	Charcoal/grill	587	170.7410	52.5773	ND	1000
13	Pyrene (Pyr)	HPLC-FLD/ GC–MS/ HPLC-DAD	Charcoal/grill	603	359.3156	93.9472	ND	1888
14	Anthracene (Anth)	HPLC-FLD/ GC–MS/ HPLC-DAD	Charcoal/grill	620	83.9993	9.8451	ND	157.5
15	Fluoranthene (Flrt)	HPLC-FLD/ GC–MS/ HPLC-DAD	Charcoal/grill	630	91.7564	39.6825	ND	701
16	Aceaphthene (Ace)	HPLC-FLD/ GC–MS/ HPLC-DAD	Charcoal/grill	559	3.6591	0.7860	ND	26.17

1 High performance liquid chromatography with fluorescence detector (HPLC*/*FLD).

2 Gas chromatography–mass spectrometry (GC–MS).

3 High-performance liquid chromatography with diode-array detection (HPLC-DAD).

4 Not Detected (ND).

### Health risk assessment

#### Health risk estimation by MCS

This study aimed to assess the potential health risks associated with the consumption of meat kebabs by calculating and simulating the concentration of PAHs. Table 3 shows the outcomes of 10,000 MCS trials. It shows important numbers like the mean, the standard deviation, and other factors for CDI that are related to both cancer-causing and non-cancerous risks. The mean values of CDI for BaP, Anth, Ace, Acy, Pyr, Flrt, Flu, and Nap in meat were 3.65 × 10^−8^ and 3.60 × 10^−2^, 3.20 × 10^−10^ and 3.23 × 10^−2^, 1.10 × 10^−11^ and 1.10 × 10^−2^, 8.15 × 10^−11^ and 8.14 × 10^−2^, 8.56 × 10^−11^ and 8.69 × 10^−2^, 6.80 × 10^−11^ and 6.75 × 10^−2^, 8.76 × 10^−11^ and 8.88 × 10^−2^, 6.76 × 10^−11^ and 6.79 × 10^−2^ mg/kg.day respectively.

**Table 3:** Probability assessment of CDI (mg/kg.day) due to PAHs in meat kebabs.

**PAHs**	**Risk**	**Min**	**Max**	**Mean**	**SD**	**10 %**	**30 %**	**60 %**	**90 %**
**BaP**	carcinogenic	3.41E-9	1.68E-7	3.65E-8	1.88E-8	1.60E-8	2.48E-8	3.74E-8	6.25E-8
	non-carcinogenic	2.94E-3	1.78E-1	3.60E-2	1.90E-2	1.55E-2	2.43E-2	3.70E-2	6.10E-2
**Anth**	carcinogenic	8.91E-11	3.20E-9	3.20E-10	2.41E-10	1.48E-10	1.95E-10	2.83E-10	5.63E-10
	non-carcinogenic	8.44E-3	3.59E-1	3.23E-2	2.47E-2	1.49E-2	1.97E-2	2.86E-2	5.66E-2
**Ace**	carcinogenic	5.04E-13	7.73E-11	1.10E-11	6.75E-12	4.39E-12	6.96E-12	1.09E-11	1.94E-11
	non-carcinogenic	4.69E-4	5.77E-2	1.10E-2	6.77E-3	4.44E-3	6.91E-3	1.09E-2	1.93E-2
**Acy**	carcinogenic	1.56E-10	2.13E-10	8.15E-11	3.72E-11	3.48E-11	6.68E-11	9.29E-11	1.24E-10
	non-carcinogenic	1.70E-1	2.00E-1	8.14E-2	3.70E-2	3.51E-2	6.69E-2	9.29E-2	1.24E-1
**Pyr**	carcinogenic	7.66E-12	4.81E-10	8.56E-11	4.75E-11	3.86E-11	5.68E-11	8.54E-11	1.44E-10
	non-carcinogenic	9.97E-3	4.88E-1	8.69E-2	4.92E-2	3.89E-2	5.72E-2	8.65E-2	1.47E-1
**Flrt**	carcinogenic	1.27E-11	4.51E-10	6.80E-11	3.71E-11	3.28E-11	4.56E-11	6.68E-11	1.15E-10
	non-carcinogenic	1.20E-2	5.24E-1	6.75E-2	3.71E-2	3.22E-2	4.55E-2	6.62E-2	1.13E-1
**flu**	carcinogenic	3.84E-12	3.73E-10	8.76E-11	4.12E-11	4.19E-11	6.29E-11	9.05E-11	1.42E-10
	non-carcinogenic	1.93E-3	4.21E-1	8.88E-2	4.17E-2	4.27E-2	6.37E-2	9.22E-2	1.43E-1
**Nap**	carcinogenic	6.20E-12	5.04E-10	6.76E-11	4.47E-11	2.66E-11	4.10E-11	6.52E-11	1.23E-10
	non-carcinogenic	6.36E-3	5.39E-1	6.79E-2	4.38E-2	2.65E-2	4.16E-2	6.63E-2	1.22E-1

#### Non-carcinogenic risk assessment

**Fig. 2:**
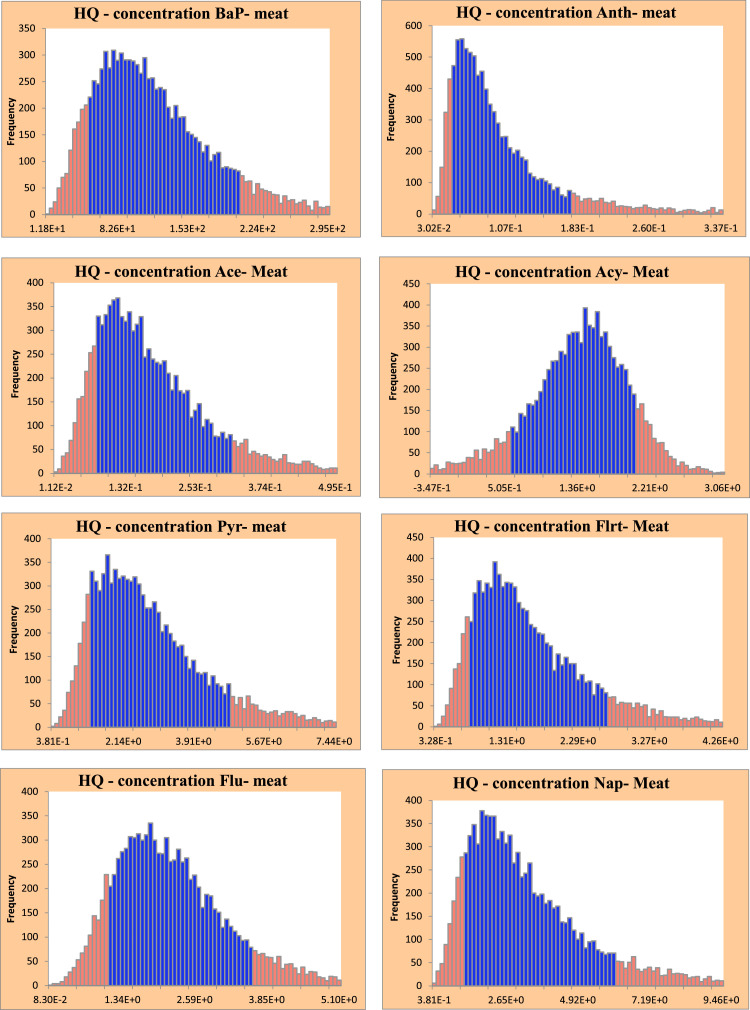
Histogram of the HQ values for PAHs in meat kebabs.

Table 4 displays a comprehensive assessment of probability HQ associated with PAHs in meat kebabs, encompassing key statistical parameters such as the minimum, maximum, average, and standard deviation, as well as the percentiles at the 10th, 30th, 60th, and 90th levels. According to Table 4, the mean values of HQ in Bap, Anth, Ace, Acy, Pyr, Flrt, Flu, and Nap in meat were 1.20 × 10^+2^, 1.08 × 10^−1^, 1.83 × 10^−1^, 2.84, 2.90, 1.69 and 2.22, respectively. Moreover, the mean values of HQ for PAHs were greater than one. In Fig. 2, the distributions of HQ levels for PAHs in meat are presented. Based on the data depicted in Fig 2, the HQ levels for the 10th and 90th percentiles of Bap, Anth, Ace, Acy, Pyr, Flrt, Flu, and Nap range from 5.16 × 10¹ to 2.03 × 10², 4.96 × 10⁻² to 1.89 × 10⁻¹, 7.39 × 10⁻² to 3.21 × 10⁻¹, 5.85 × 10⁻¹ to 2.06, 1.30 to 4.92, 8.05 × 10⁻¹ to 2.83, 1.07 to 3.58, and 1.32 to 6.10, respectively.

**Table 4:** Probability assessment of HQ due to PAHs in meat kebabs.

**PAHs**	**Min**	**Max**	**Mean**	**SD**	**10 %**	**30 %**	**60 %**	**90 %**
**BaP**	9.79E+0	5.95E+2	1.20E+2	6.32E+1	5.16E+1	8.09E+1	1.23E+2	2.03E+2
**Anth**	2.81E-2	1.20E+0	1.08E-1	8.25E-2	4.96E-2	6.56E-2	9.52E-2	1.89E-1
**Ace**	7.82E-3	9.61E-1	1.83E-1	1.13E-1	7.39E-2	1.15E-1	1.81E-1	3.21E-1
**Acy**	1.36E+0	3.34E+0	2.84E+0	6.17E-1	5.85E-1	1.11E+0	1.55E+0	2.06E+0
**Pyr**	3.32E-1	1.63E+1	2.90E+0	1.64E+0	1.30E+0	1.91E+0	2.88E+0	4.92E+0
**Flrt**	3.01E-1	1.31E+1	1.69E+0	9.27E-1	8.05E-1	1.14E+0	1.65E+0	2.83E+0
**flu**	4.82E-2	1.05E+1	2.22E+0	1.04E+0	1.07E+0	1.59E+0	2.31E+0	3.58E+0
**Nap**	3.18E-1	2.70E+1	3.39E+0	2.19E+0	1.32E+0	2.08E+0	3.32E+0	6.10E+0

#### Carcinogenic risk assessment

Table 5 provides a full and detailed explanation of the estimated LTCR from PAHs in meat kebabs. The results come from a large dataset of 10,000 trials. This presentation encompasses a diverse set of crucial statistical metrics, including the average (mean), standard deviation, minimum and maximum values, and percentile values at the 10th, 30th, 60th, and 90th percentiles. According to Table 5, the mean values of LTCR in Bap, Anth, Ace, Acy, Pyr, Flrt, Flu, and Nap were 3.65 × 10^−8^, 3.20 × 10^−10^, 1.10 × 10^−11^, 8.15 × 10^−11^, 8.56 × 10^−11^, 6.80 × 10^−11^, 8.76 × 10^−11^ and 6.76 × 10^−11^, respectively.

**Table 5:** Probability assessment of LTCR due to PAHs in meat kebabs.

**PAHs**	**Min**	**Max**	**Mean**	**SD**	**10 %**	**30 %**	**60 %**	**90 %**
**BaP**	3.41E-9	1.68E-7	3.65E-8	1.88E-8	1.60E-8	2.48E-8	3.74E-8	6.25E-8
**Anth**	8.91E-11	3.20E-9	3.20E-10	2.41E-10	1.48E-10	1.95E-10	2.83E-10	5.63E-10
**Ace**	5.04E-13	7.73E-11	1.10E-11	6.75E-12	4.39E-12	6.96E-12	1.09E-11	1.94E-11
**Acy**	3.72E-12	2.13E-10	8.15E-11	3.72E-11	3.48E-11	6.68E-11	9.29E-11	1.24E-10
**Pyr**	7.66E-12	4.81E-10	8.56E-11	4.75E-11	3.86E-11	5.68E-11	8.54E-11	1.44E-10
**Flrt**	1.27E-11	4.51E-10	6.80E-11	3.71E-11	3.28E-11	4.56E-11	6.68E-11	1.15E-10
**Flu**	3.84E-12	3.73E-10	8.76E-11	4.12E-11	4.19E-11	6.29E-11	9.05E-11	1.42E-10
**Nap**	6.20E-12	5.04E-10	6.76E-11	4.47E-11	2.66E-11	4.10E-11	6.52E-11	1.23E-10

**Fig. 3:**
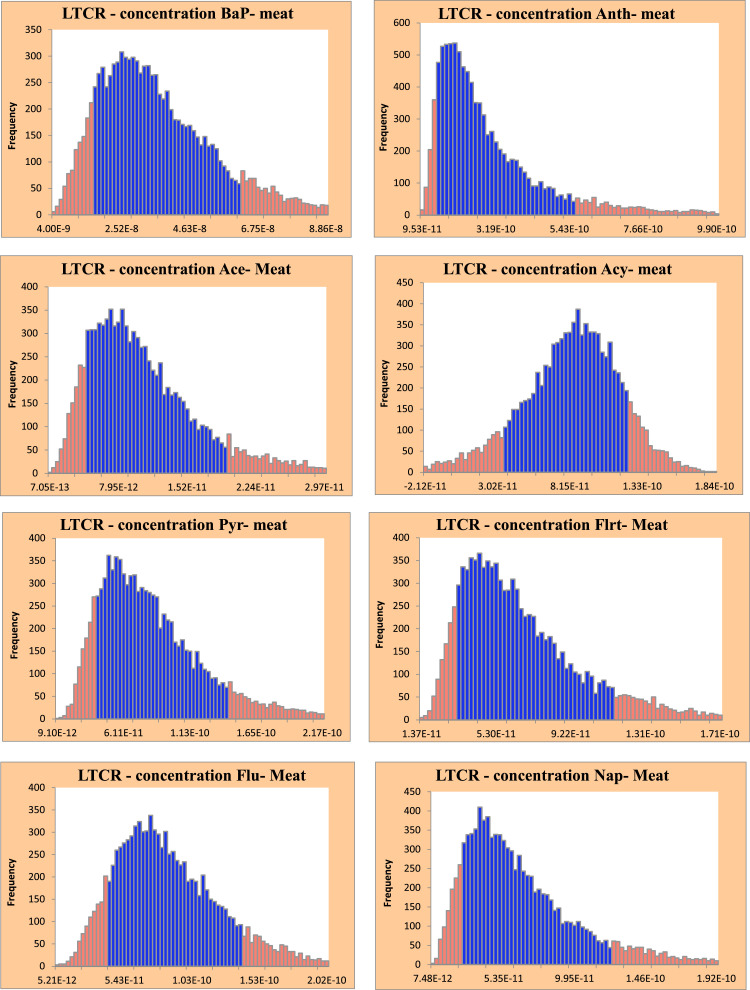
Histogram of the LTCR values for PAHs in meat kebabs.

Fig. 3 shows the full range of LTCR values connected with PAHs found in meat kebabs, giving a thorough picture of the possible health risks. The data in Fig. 3 shows that the LTCR values for the 10th and 90th percentiles of Bap, Anth, Ace, Acy, Pyr, Flrt, Flu, and Nap were 1.60 × 10^−8^ to 6.25 × 10^−8^, 1.48 × 10^−10^ to 5.63 × 10^−10^, 4.39 × 10^−12^ to 1.94 × 10^−11^, 3.48 × 10^−11^ to 1.24 × 10^−10^, 3.86 × 10^−11^ to 1.44 × 10^−10^, 3.28 × 10^−11^ to 1.15 × 10^−10^, 4.19 × 10^−11^ to 1.42 × 10^−10^, and 2.66 × 10^−11^ to 1.23 × 10^−10^. These findings provide valuable insights into the potential cancer risks associated with exposure to these PAHs at different levels.

## Discussion

PAHs constitute a category of pervasive and persistent organic contaminants, widely distributed in the environment, encompassing air, water, soil, sediment, and diverse food sources [22]. Meat products, especially those rich in fat, are a significant contributor to the intake of PAHs. Food's PAH content directly correlates with its lipid content. When meat comes into direct or indirect contact with flames, the fat inside the meat breaks down, creating PAHs that are then transferred to the meat. Food products are essential in disease management, especially for cancer prevention [[23], [24], [25]]. After a careful study of the research and literature, it was found that the amount of heat used, the type of heat source used, and the specific techniques used to prepare meat products have a big impact on the production and concentration of PAHs in these products. Among the various culinary techniques employed in cooking, grilling stands out, particularly the method of direct charcoal grilling. This specific technique has consistently shown a significantly higher probability for the formation of PAHs. This phenomenon occurs primarily due to the combustion of fats and the subsequent exposure of food to smoke generated during the grilling process. In contrast, alternative cooking techniques like frying, roasting, or boiling typically yield much lower levels of PAHs.

Boiling, for example, utilizes indirect heat within a water environment and does not encourage the pyrolysis of organic substances, leading to minimal PAH formation [26,27]. Frying may lead to the creation of PAHs, especially at high temperatures and in reused oils, however, the concentrations are generally less than those found in open-flame grilling [6]. Roasting and baking in the oven produce reduced levels of PAHs in comparison to grilling, as long as the cooking temperature is regulated and fat does not fall onto the heating elements. These differences emphasize the importance of cooking method selection in minimizing dietary PAH exposure [28,29].

Table 4, Table 5 indicate that this study meticulously examined several meat products for both carcinogenic and non-carcinogenic PAHs. Our investigation revealed that the concentration of PAHs exhibited significant variability across a range of meat cooking and preparation methods. Significant regional differences in PAH levels in meat products have been documented, primarily shaped by variations in cooking methods, types of fuel used, and the enforcement of regulations. Nations that implement strict food safety regulations, like those in the EU, which impose upper thresholds of 5 µg/kg for BaP and 30 µg/kg for ∑PAHs, generally report lower contamination levels in meat samples [9,30]. In contrast, higher levels are often found in regions where traditional methods such as direct charcoal grilling are predominant and regulatory oversight is limited, including parts of Asia, Africa, and the Middle East [31,32]. Moreover, advanced cooking techniques, including indirect heating, gas, or electric grilling, have demonstrated a notable decrease in PAH production by limiting fat combustion and smoke exposure [29,33]. For example, Ross et al. (2015) demonstrated that preventing fat drippings onto heat sources through proper grill design effectively reduces PAH formation in grilled meat products [28]. The average concentration of HQ in Bap, Nap, and Pyr was higher compared to the other five PAHs found in meat. The specific order of the average HQ values for each PAH was as follows: BaP > Nap > Pyr > Acy > Flrt > flu > Ace > Anth. Also The average concentration of LTCR in Bap, Anth, and flu was higher compared to the other 5 PAHs found in meat. The specific order of the average LTCR values for each PAH was as follows: BaP > Anth > flu > Pyr > Acy > Flrt > Nap > Ace. Olatunji, Fatoki, Opeolu, and Ximba (2014) found high levels of benzo[k]fluoranthene, BaP, indeno[123-cd]pyrene, and benzo[ghi]perylene in heat-processed meat in South Africa. These levels ranged from 0.07 to 46.67 ng/g [33]. The variations in PAH concentrations in heat-treated meat can be ascribed to several factors, including the meat's distance from the fire, cooking temperature, cooking duration, type of meat cut, and fat content. Thus, by adeptly regulating these parameters, one can reduce the generation of PAHs in heat-processed meat and meat products [26,33]. In general, PAH consumption through diet relies on the dietary patterns of the local populace and the contamination levels of PAHs in food. Thus, those who frequently ingest large quantities of meat products may encounter markedly elevated daily exposure to PAHs compared to the general population [34]. Numerous alternative cooking techniques have demonstrated efficacy in reducing the formation of PAHs in meat. Indirect grilling, which involves cooking meat adjacent to the heat source rather than directly above it, minimizes direct fat combustion and smoke exposure, significantly lowering PAH levels compared to direct grilling methods [29]. Additionally, marinating with ingredients abundant in antioxidants like lemon juice, garlic, tea, and rosemary has been demonstrated to reduce PAH formation by neutralizing free radicals and restricting lipid oxidation during thermal processing [35,36]. Cooking techniques that employ lower temperatures, such as slow roasting or sous-vide followed by a brief sear, mitigate pyrolysis reactions and thereby reduce the formation of PAHs. These methods represent effective strategies to diminish dietary exposure to carcinogenic compounds while preserving the quality and flavor of the meat [27,37]. The EPA's Exposure Factors Handbook indicates that the average life expectancy of the general population is estimated at 70 years, which is utilised to represent ED. Cancerous effects can arise at any point in a person's life as a result of the cumulative exposures to risk factors they experience. In contrast, non-cancerous effects only occur during exposure. Consequently, it is important to note that the average time for the manifestation of cancerous effects differs from the average time for the manifestation of non-cancerous effects [18,38]. Based on the data presented in Table 4, it can be observed that Anth had the lowest average non-carcinogenic risk (HQ) of 1.08 × 10^–1^, while Bap had the highest average non-carcinogenic risk (HQ) of 1.20 × 10^2^. Additionally, as shown in Table 5, it is clear that Ace recorded the lowest average LTCR (1.10 × 10–11), while Bap displayed the highest average LTCR,(3.65 × 10–8). The HQ values in this study were interpreted based on USEPA guidelines, where an HQ > 1 indicates a potential non-carcinogenic health risk due to chronic exposure, whereas an HQ < 1 suggests negligible risk. In our analysis, some PAHs such as BaP and Nap exhibited HQ values exceeding 1, implying a potential for non-carcinogenic effects under high exposure scenarios. According to the USEPA, an LTCR below 1 × 10⁻⁶ is considered negligible, while values between 1 × 10⁻⁶ and 1 × 10⁻⁴ fall within an acceptable risk range. An LTCR exceeding 1 × 10⁻⁴ may be of regulatory concern and could warrant further risk management interventions [39,40]. All calculated LTCR values in this study, including those for BaP, Anth, and Flu, remained below 1 × 10⁻⁶, suggesting that typical consumption of meat kebabs does not pose a significant carcinogenic risk. Therefore, while non-carcinogenic risks for specific PAHs may exist, the overall cancer risk from dietary exposure to PAHs in meat kebabs appears to be within acceptable safety margins for the general population. The study found that the levels of PAHs in grilled meats were significantly higher when cooked using coal fuel as compared to gas fuel, across all types of meats. The reason for this difference might be that charcoal doesn't burn completely like gas fuel does, so it makes soot compounds that are high in PAHs [29]. Additionally, many factors can influence the formation of PAHs during the charcoal broiling of meat. Some of these factors include the amount of fat content in the meat, distance of heat source from meat, and exposure duration of meat to heat. When grilling meat, the fat drips into the flames, producing smoke that contains different compounds, including PAHs [27,41]. The research conducted by Ross et al. (2015) reveals that when appropriate measures are implemented to prevent the dripping of melted fat onto the heat source, such as utilising properly designed grills or an electric grill, grilled products are devoid of any carcinogenic PAHs [28]. Several epidemiological studies have established that excessive consumption of meat, particularly red meat cooked at high temperatures, is associated with increased risk of rectal and colon cancers. Furthermore, estimates project that food components are accountable for over 80 % of all observed incidences of colorectal cancer [31]. For example, a case-control study conducted at Sultan Qaboos University Hospital, Oman (2018) showed a significant correlation between the incidence of colorectal cancers and lower consumption of dietary fibre, and more consumption of meat [42]. Further, the study by Buamden (2018) reported that Uruguay, Barbados, Argentina, and Cuba reported the highest incidence of colorectal cancer in the Americas. These cases were strongly linked to the consumption of animal fat and red meat, which has been shown to contribute to the formation of carcinogenic PAHs [43]. Also, in Table 2, the average LTCR for all PAHs is within an acceptable and inconsequential level (LTCR = 10^−6^). Therefore, there is no significant possibility of carcinogenic effects resulting from the presence of PAHs when consuming meat. The studies have revealed that altering the cooking method has a considerable effect on the creation and concentration of PAHs. The foods that underwent flame grilling and charcoal grilling processes exhibited a substantial increase in the formation of PAHs, with levels reaching as high as 350 mg/kg. This phenomenon is likely attributed to the deposition of PAHs resulting from smoke exposure during the grilling process [44].

After conducting a thorough comparison of the BaP concentrations and other PAH levels between direct charcoal-grilled chicken, beef, and pork, and indirect charcoal grilling, a notable discovery was made: the application of direct charcoal grilling resulted in a minimum fivefold increase in these substances [45]. For example, an comprehensive study on barbecuing in the UK (2015) using a range of types of meat (beef, lamb, salmon, chicken, pork, and sausage) and heating methods and sources of energy (charcoal, gas, charcoal with wood chips, and briquettes) systematically reported comparable trends [28]. Gorji et al. (2016) evaluated 16 PAHs in four distinct types of kebabs in Tehran, Iran. The average amount of total PAHs (PAHs) in the samples changed from 7.37 to 17.94 µg/kg, and the amount of BaP changed from 0.28 to 5.81 µg/kg [32]. The cooking level significantly influences the development of PAHs in cooked meat [46]. Hamidi et al. (2022) used charcoal to grill chicken and beef meat samples at three levels: well-done, medium-done, and rare. They did this to see how the level of cooking affects the bioavailability of PAHs. The study found that the bioaccessibility of the analyzed PAHs varied depending on the level of grilling in the beef and chicken samples. The bioaccessibility ranged from 6.51 % to 84.83 %, 5.27 % to 63.55 %, and 2.38 % to 43.75 % for well-done, medium, and rare grilled beef, respectively. Similarly, the bioaccessibility of well-done, medium, and rare grilled chicken was 2.95 % to 87.77 %, 1.30 % to 32.50 %, and 0.67 % to 17.33 %, respectively. The findings of this study show that the average bioaccessibility of PAHs from the meat matrix goes up as the desired level of doneness goes up [47]. Moreover, this study investigated the influence of different heat sources on PAH formation in meat. In particular, 63.2 % of the meat samples were cooked by charcoal heat, and 36.8 % were cooked by gas heat. In addition, the studies reviewed herein were clearly indicative of a significant decrease in PAH levels when the source of heat was changed from coal to gas [32]. The study by Chung et al. (2011) found that 0.03 μg/kg of PAHs were found in gas-roasted beef samples, 0.78 μg/kg were found in charcoal-grilled beef samples, and 0.03 μg/kg were found in charcoal-roasted beef samples. The difference observed between gas and coal flames can be explained by the different natures of the thermal energy that they produce. The production of drier heat and higher temperatures distinguishes coal flames from gas flames [29]. Additionally, the distribution of cooking methods for meats revealed that 40.8 % were prepared using indirect heat, while the majority, accounting for 59.2 %, were cooked using direct heat. In the direct cooking method, the food directly engages with a thermal source, such as a gas oven or charcoal grill. Conversely, the indirect cooking method, shown by electric oven toasting, does not entail direct contact between the food and the heat source, hence producing an indirect effect [48]. The determination of PAHs in food matrices has been a difficult task because of the food matrix character and the fact that PAHs exist in trace amounts in the matrices. The application of sophisticated analysis instruments, where methods such as HPLC, GC, and fluorescence or mass spectrometry detection were utilized, greatly improved the sensitivity and accuracy of PAH determination in food samples [49]. In the present study, 33.4 % of the samples were examined using HPLC, and 59.2 % using GC procedures. Nevertheless, it can be pointed out that these conventional techniques use large amounts of solvents. Increasing awareness of the role PAHs play in human health has helped to advance the techniques of extraction and quantitation of these compounds in food products. According to epidemiologists, a significant correlation exists between the consumption of foods that contain PAHs and the development of various types of cancer. Hence, it is of utmost importance to discover and implement effective methods for preventing the formation of PAHs during the process of meat processing. A lot of studies have shown that using certain marinades with antioxidant-rich ingredients like garlic, onions, lemon, and tea can greatly reduce the production and buildup of PAHs. Antioxidants are widely recognized for their remarkable ability to neutralize and eliminate harmful free radicals, which can cause oxidative stress and damage to cells and tissues in the body [[35], [36], [37]].

According to the results of this research, various practical public health suggestions can be proposed to decrease dietary intake of PAHs from grilled meats. Initially, consumers should adopt indirect grilling techniques or utilize gas/electric grills rather than open-flame charcoal grills to lessen smoke and fat burning. Marinating meat with antioxidant-rich ingredients (e.g., lemon, garlic, tea) before cooking can also inhibit PAH formation. Furthermore, it is essential to prevent overcooking, charring, and direct exposure of fat to flames. Public health officials should prioritize the development or enhancement of national standards regarding safe grilling practices and temperature regulation during the cooking process. Regulatory agencies must continue to monitor the concentrations of PAHs in commercially available food as well as in street food. Additionally, it is crucial to establish or standardize the maximum permissible levels of PAHs in heat-processed meat products. These actions would promote informed consumer behaviors and enhance cancer prevention initiatives by minimizing exposure to chemical hazards in food.

## Conclusion

This study looked at 16 PAHs in meat kebabs via a systematic review and meta-analysis methodology. The study aimed to determine how the compounds can impact human health, both in non-carcinogenic and carcinogenic risks. According to the study's findings, the concentration of PAHs in meat kebabs was found to vary significantly depending on the cooking method employed, with grilling being identified as the most frequently utilized technique. Risk analysis and a detailed study of the research found a strong link between the consumption of grilled meat and a high chance of cancer development. This is mainly due to the generation of harmful compounds, especially BaP. The HQ for PAHs in daily meat kebabs showed that there is no clear cancer risk from eating meat kebabs around the world, based on the EPA's recommendation of the highest acceptable risk level. In summary, this study highlights the need to recognise the health concerns linked to PAHs and stresses the need for educated choices about food consumption and preparation. The ultimate goal is to limit the intake of unhealthy chemicals like PAHs, thereby guarding our health. While this study provides a quantitative risk assessment based on pooled data, it underscores the need for long-term cohort studies and human biomonitoring efforts to evaluate the chronic health impacts of dietary PAH exposure. Existing epidemiological evidence linking PAH intake to cancer and other diseases is largely extrapolated from occupational or environmental exposure settings, and few large-scale prospective studies have focused specifically on foodborne PAHs. Given the popularity of grilled meat dishes such as kebabs in many cultures, there is a critical need to design longitudinal studies that monitor dietary habits, PAH biomarkers (e.g., urinary 1-hydroxypyrene), and associated health outcomes over time.

## CRediT author statement

Hadi Niknejad: Writing- Reviewing and Editing, Supervi-sion, Design of study; Fatemeh Mortezazadeh: Data curation, Writing – original draft, Methodology; Somayeh Hoseinvandtabar: Writing- Original draft preparation, Data curation; Anoushiravan Mohseni-Bandpei: Software, Writing- Reviewing and Editing; Fathollah Gholami-Borujeni: Software, Conceptualization, Supervision, Methodology.

## Financial interests

The authors declare they have no financial interests.

## Declaration of competing interest

The authors declare that they have no known competing financial interests or personal relationships that could have appeared to influence the work reported in this paper.
